# Selenoproteins—Tracing the Role of a Trace Element in Protein Function

**DOI:** 10.1371/journal.pbio.0030421

**Published:** 2005-12-13

**Authors:** Thressa C Stadtman

## Abstract

Selenium is an important component of several enzymes, replacing sulfur in cysteine residues. Its discovery and significance are described in this primer.

In retrospect, the history of selenium biochemistry does not differ greatly from the study of a number of other trace elements in that it occurred over many years, progressing from periods of little general interest to widespread concern regarding toxicity problems and eventually to recognition of selenium as an essential nutrient for many forms of life. My involvement in studies on selenium metabolism is a classic example of serendipity. I was studying an interesting enzyme from an anaerobic bacterium that utilized glycine as substrate (glycine reductase), but the amounts of pure protein I could isolate were very limited because it seemed to be produced only during the very early stages of bacterial cell growth. The rich culture medium supported continued luxuriant growth of the bacterium, but the level of the desired enzyme in the cells merely underwent dilution during the process. Finally, after testing many known growth stimulatory supplements to no avail, my colleagues and I tried two inorganic nutrients, molybdate and selenite. This approach was prompted by the report that addition of these inorganic compounds to a medium used for anaerobic growth of Escherichia coli supported synthesis of the enzyme formate dehydrogenase [1]. Much to my delight, addition of selenite to our growth medium resulted in greatly increased levels of the glycine reductase enzyme, and synthesis of the protein continued throughout the entire growth period. Thus, a common belief among microbiologists that a so-called rich culture medium high in tryptone and yeast extract content was nutritionally adequate is incorrect if growth of the organism depends on ability to synthesize a selenium-containing enzyme. In our case other amino acids in the medium could support growth even when the trace of selenium was depleted and synthesis of glycine reductase stopped. With this unexpected finding, we had an ideal biological system for unraveling details of the role of selenium in the anaerobic metabolism of glycine.

The element selenium was discovered by the Swedish chemist Berzelius in 1817, and the few organic selenium-containing compounds that were prepared during the subsequent 100 years were considered primarily as chemical curiosities. Finally, in the 1930s, selenium was recognized as the potent toxic substance present in various types of plants that, when ingested by grazing animals, caused chronic symptoms of poisoning. Determination of the selenium contents of several native plants from Wyoming and South Dakota revealed that members of the genus Astragalus accumulated extremely high levels of selenium, as much as several thousand parts per million. During periods of drought, when appreciable amounts of these selenium accumulator plants were consumed in spite of their unpleasant odor, animals exhibited symptoms of acute poisoning, such as “blind staggers,” loss of appetite, paralysis, and finally death. In the arid regions of the western United States, selenium accumulation in soils is much higher than in areas that have normal rainfall, and as a result even ordinary plants, such as cereal grains, from this area contain unusually high levels of selenium. From 1930 to the mid-1950s, many investigators attempted to determine the chemical form(s) of the selenium present in the toxic plants, and animal nutritionists tested the effects of various inorganic and a few organic selenium compounds administered to animals. However, beyond the observation that much of the selenium in plant materials was protein-bound, actual identification of the toxic selenium compounds present was not accomplished [2]. In retrospect, this is not surprising because, as my coworkers and I learned firsthand much later, selenium-containing amino acids in proteins are notoriously unstable, especially when exposed to oxygen during isolation procedures. It took many years for scientists to learn how to identify and determine quantitatively the organic selenium compounds normally present in biological materials, and prior to the mid-1950s, these compounds, especially in selenium accumulator plants, were known only for their acute toxic effects. As is the case with other elements known originally only as toxins and later shown to be required by particular living organisms, selenium eventually was found to be an essential nutrient for animals and several species of bacteria.

In 1957, Klaus Schwarz, a German scientist working at the US National Institutes of Health in Bethesda, Maryland, reported that selenium was the essential component of a dietary preparation termed Factor 3 that prevented severe liver necrosis in rats [3]. Factor 3 preparations isolated from brewer's yeast and from casein were especially effective sources of the active selenium component. During this same period, an important disease in young chickens and turkeys known as exudative diathesis was recognized by Nesheim and Scott [4] as a symptom of selenium deficiency. Administration of Factor 3 or inorganic forms of selenium prevented development of the deficiency syndrome. However, the basic defect responsible for the selenium deficiency symptoms remained undefined for several more years.

Finally, in 1973, it was reported that the catalytic activities of two different enzymes depended on the presence of selenium in these proteins. One of these enzymes was my favorite glycine reductase from anaerobic bacteria [5], and the other was mammalian glutathione peroxidase, studied by investigators at the University of Wisconsin [6]. It was shown that when radioactive selenium (^75^Se) was provided, it was incorporated into both proteins during in vivo synthesis. After much effort spent in learning how to handle oxygen-sensitive selenium compounds, we finally could identify the selenium compound in our glycine reductase protein as selenocysteine, an analog of the sulfur-containing amino acid cysteine [7]. The selenocysteine occurs at a specific position in the protein polypeptide chain. Employing our methods for selenocysteine identification, Tappel and coworkers [8] at the University of California at Davis showed that the essential selenium in glutathione peroxide was also present as a selenocysteine residue in the protein.

Eventually scientists began to understand how proteins are built in living organisms and how the exact location of a naturally occurring amino acid in a protein is determined by the genetic code, but the inclusion of unusual amino acids remained a matter of speculation. Largely through the efforts of a young German scientist named August Böck and his students at the University of Munich, the question of how an amino acid such as selenocysteine could be inserted in a highly specific location in a protein could be answered. The first report in 1986 was followed by an impressive series of investigations dealing with the biosynthesis of selenoproteins in E. coli (reviewed in [9]). As it turned out, a sequence in the nucleic acid message that normally told the protein-synthesizing machinery to terminate the process—the stop codon UGA—now triggered a different set of instructions, namely, to direct selenocysteine insertion at the UGA codon. To achieve this reprogramming, cells have acquired an impressive number of additional chemical steps. A new amino acid transfer nucleic acid (tRNA) that could read the UGA codon was discovered. Selenocysteine derived from a serine initially bound to this tRNA is inserted into a growing protein chain. In E. coli, the selenium donor required for conversion of the serine on the tRNA to selenocysteine proved to be selenophosphate, an energy-rich compound in which selenium is bonded directly to the phosphorus atom [10]. This oxygen-labile selenium compound is known to be produced by a number of mammals and bacteria that synthesize specific selenoenzymes, and, based on present knowledge, a biological role for this reactive selenium compound as a “universal selenium donor” may be correct.

A new paper in this issue of *PLoS Biology* addresses the question of why selenium substitution for sulfur at the active site of an enzyme can be an advantage and in many cases has been preserved in nature [11]. Kim and Gladyshev address the catalytic mechanism of mammalian methionine-*R*-sulfoxide reductase (MsrB) 1, which contains selenocysteine at the active site, and compare it to the catalytic mechanism of the two other forms of the mammalian enzyme (MsrB2 and MsrB3) in which cysteine is found instead. Reduction of the methionine sulfoxide substrate to methionine by the active site cysteine residue in MsrB2 or MsrB3 converts the cysteine thiol to a sulfenic acid derivative (SOH), which can be reduced by thioredoxin to regenerate cysteine. In contrast, reduction of the sulfoxide substrate by MsrB1 converts the ionized selenocysteine residue to a selenenic acid derivative (SeOH), which is not reduced directly by thioredoxin (Figure 1). However, regeneration of MsrB1 by thioredoxin can occur after the SeOH group is converted to a selenosulfide intermediate by reaction with a unique cysteine residue in MsrB1.

**Figure 1:**
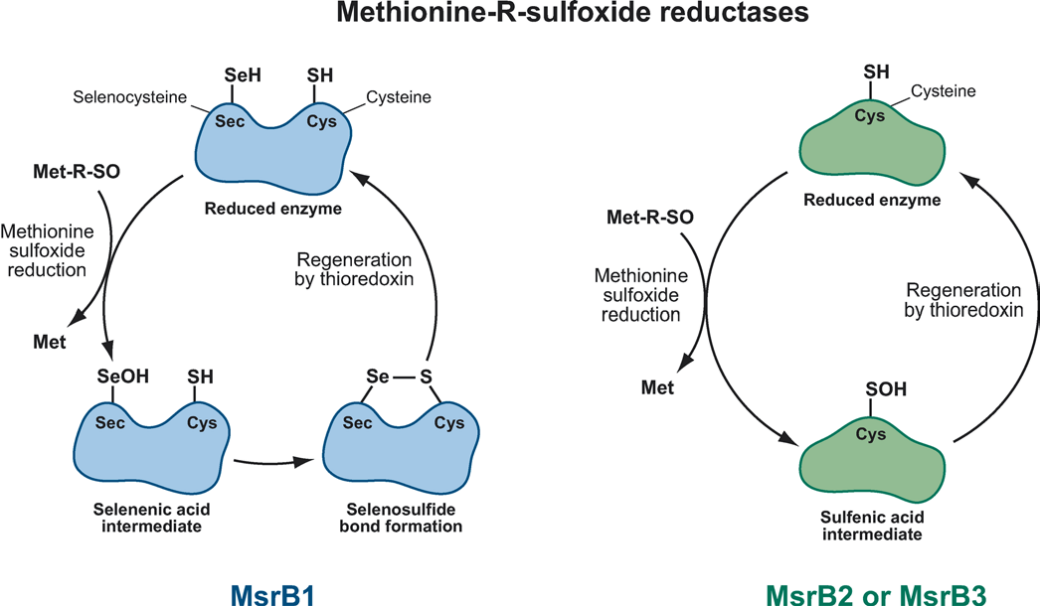
Schematic Illustrations Comparing the Enzymatic Reactions of Three Mammalian MsrBs MsrB1 is a selenium-containing protein, whereas MsrB2 and MsrB3 contain cysteine in the active site. (Illustration: Rusty Howson, sososo design)

An impressive number of mutant forms of the genes corresponding to the three MsrB enzymes were constructed, cloned, and expressed in E. coli, and the catalytic activities and substrate affinities of the purified gene products were determined in detail by the authors. The native MsrB2 and MsrB3 enzymes contain three highly conserved amino acid residues—namely, His-77, Val- or Ile-81, and Asn-97—that are part of the active sites of these enzymes. Completely different amino acid residues, Gly-77, Glu-81, and Phe-97, are found at these positions in the fully active form of MsrB1. A series of mutant constructs in which the amino acids at positions 77, 81, and 97 were switched individually or in groups between native selenocysteine and cysteine forms of MsrB, and also between selenocysteine- or cysteine-substituted enzymes, were analyzed in detail. Several of these mutants exhibited marked changes in the ability to utilize the normal electron donor, thioredoxin, for enzyme regeneration. Replacement of the active site selenocysteine in MsrB1 with cysteine resulted in greatly decreased catalytic activity, which could be partially restored by introduction of His-77 and Asn-97 at the active site. Mutants that were modified extensively to allow insertion of selenocysteine at the active site in place of cysteine were also generated, and their characteristics were determined. In general, the replacement of cysteine with selenocysteine frequently resulted in increased activity with dithiothreitol as reductant, but regeneration of active enzyme in these constructs by the natural electron donor, thioredoxin, was not possible. Based on the reported findings, it is clear that different critical amino acids in the active sites of the selenocysteine and cysteine enzymes are required for their maximal catalytic activities, and these also determine electron donor specificity for enzyme turnover.

The last few years have seen the emergence of numerous important physiological roles for the trace element selenium. In contrast, it took many years from Berzelius's discovery of this new element in 1817, which he named after Selene, the goddess of the moon in ancient Greece, until it attracted growing interest, whereupon it gained a bad reputation as a toxic substance. Even after its recognition as a required nutrient for mammals, the role of selenium as an essential component of important antioxidant enzymes synthesized in our cells is not widely appreciated. Further studies of the unique properties of selenium will help us to understand the selective advantage imparted to cells by their investment in selenoprotein biosynthesis and its retention during evolution.

## Abbreviations

MsrB: methionine-*R*-sulfoxide reductase

## References

[pbio-0030421-b1] Lester RL, DeMoss JA (1971). Effects of molybdate and selenite on formate and nitrate metabolism in Escherichia coli. J Bacteriol.

[pbio-0030421-b2] Stadtman TC (1974). Selenium biochemistry. Science.

[pbio-0030421-b3] Schwarz K, Foltz CM (1957). Selenium as an integral part of Factor 3 against dietary necrotic liver degeneration. J Am Chem Soc.

[pbio-0030421-b4] Nesheim MC, Scott ML (1961). Nutritional effects of selenium compounds in chicks and turkeys. Fed Proc.

[pbio-0030421-b5] Turner DC, Stadtman TC (1973). Purification of protein components of clostridial glycine reductase system and characterization of protein A as a selenoprotein. Arch Biochem Biophys.

[pbio-0030421-b6] Rotruck JT, Pope AL, Ganther HE, Swanson AB, Hafeman DG (1973). Selenium: Biochemical role as a component of glutathione peroxidase. Science.

[pbio-0030421-b7] Cone JE, Martin del Rio R, Stadtman TC (1976). Chemical characterization of the selenoprotein component of clostridial glycine reductase: Identification of selenocysteine as the organoselenium moiety. Proc Natl Acad Sci U S A.

[pbio-0030421-b8] Forstrom JW, Zakowski JJ, Tappel AL (1978). Identification of the catalytic site of rat liver glutathione peroxidase as selenocysteine. Biochemistry.

[pbio-0030421-b9] Böck A, Hatfield DA (2001). Selenium metabolism in bacteria. Selenium: Its molecular biology and role in human health.

[pbio-0030421-b10] Glass RS, Singh WP, Jung W, Veres Z, Scholz TD (1993). Monoselenophosphate: Synthesis, characterization, and identity with the prokaryotic biological selenium donor, compound SePX. Biochemistry.

[pbio-0030421-b11] Kim HY, Gladyshev VN (2005). Different catalytic mechanisms in mammalian selenocysteine- and cysteine-containing methionine-*R*-sulfoxide reductases. PLoS Biol.

